# The Equitable Implementation of Cystic Fibrosis Personalized Medicines in Canada

**DOI:** 10.3390/jpm11050382

**Published:** 2021-05-07

**Authors:** Genevieve Shemie, Minh Thu Nguyen, John Wallenburg, Felix Ratjen, Bartha Maria Knoppers

**Affiliations:** 1 Centre of Genomics and Medicine, Department of Human Genetics, Faculty of Medicine, McGill University, Montreal, QC H3A 0C7, Canada; genevieve.shemie@mcgill.ca (G.S.); bartha.knoppers@mcgill.ca (B.M.K.); 2 Cystic Fibrosis Canada, Toronto, ON M4P 2C9, Canada; jwallenburg@cysticfibrosis.ca; 3 Department of Pediatrics Translational Medicine, Division of Respiratory Medicine, Research Institute, The Hospital for Sick Children, University of Toronto, Toronto, ON M5G 1X8, Canada; felix.ratjen@sickkids.ca

**Keywords:** personalized medicines, cystic fibrosis, healthcare equity, access, health policy, ethics, Canada

## Abstract

This article identifies the potential sources of inequity in three stages of integrating cystic fibrosis personalized medicines into the Canadian healthcare system and proposes mitigating strategies: (1) clinical research and diagnostic testing; (2) regulatory oversight and market authorization; and (3) implementation into the healthcare system. There is concern that differential access will cast a dark shadow over personalized medicine by stratifying the care that groups of patients will receive—not only based on their genetic profiles, but also on the basis of their socioeconomic status. Furthermore, there is a need to re-evaluate regulatory and market approval mechanisms to accommodate the unique nature of personalized medicines. Physical and financial accessibility ought to be remedied before personalized medicines can be equitably delivered to patients. This article identifies the socio–ethical and legal challenges at each stage and recommends mitigating policy solutions.

## 1. Introduction

In the past 20 years, our improved understanding of the role of genetics in disease has ushered in a new era of therapies for cystic fibrosis (“CF”) patients: personalized medicine. Modulator drugs, which are “personalized” and that address discrete defects in the cystic fibrosis transmembrane conductance regulator (“CFTR”) protein, have transformed disease prognosis for some CF patients. Compared to traditional symptomatic therapies, the clinical benefit of CFTR-directed drugs relies on its efficacy in restoring the CFTR gene protein function.

Currently, four modulator combinations are approved or being considered for use in Europe, Australia, Canada and the US: KALYDECO^®^ (ivacaftor); ORKAMBI^®^ (lumacaftor/ivacaftor); SYMDEKO^®^ (tezacaftor/ivacaftor); and TRIKAFTA^®^/KAFTRIO^®^ (elexacaftor/tezacaftor/ivacaftor) [1,2,3,4]. These modulators offer treatment to CF patients with certain variants, leaving those without such variants (i.e., rare or ultra-rare variants) ineligible for CFTR modulator therapy. Concretely, about 1000 of the more than 2000 CFTR variants occur in fewer than five people—the rarity of the disease creates issues in terms of physical and financial accessibility [5]. Moreover, the cost-effectiveness of CFTR modulator drugs has been criticized as personalized medicines in CF are expensive and lifelong. For example, TRIKAFTA^®^, which is currently under review in Canada [6], has a list price of USD 311,000 per patient per year; some could argue it is difficult to justify the research funding required to serve so few future eligible patients [7].

However, it is claimed that at least 90 percent of Canadian CF patients could eventually benefit from such CFTR modulators as they improve and become the standard of care [8]. In addition to its expanding scope, the implementation of personalized medicines into healthcare systems has introduced complexities for policy and regulation. As the pipeline for novel CFTR modulator drugs continues to expand, the proper translation of personalized medicines into clinical treatments will require an analysis of the socio–ethical and legal issues to ensure equitable access without exacerbating existing disparities. This paper aims to review the potential sources of inequity throughout the life cycle of CF personalized medicines in the Canadian landscape, which include the following stages: (1) clinical research and diagnostic testing; (2) regulatory oversight; and (3) implementation into the healthcare system. We recommend some promising approaches to mitigate inequity in Canadian policy for personalized medicines for cystic fibrosis. While the approaches offered are specific to the CF context, they could be applied to other more common diseases as well.

## 2. Clinical Research and Diagnostic Testing

### 2.1. Challenges

There is concern that differential access will cast a dark shadow over personalized medicine by stratifying the care that groups of patients will receive—not only on the basis of their genetic profiles, but also on the basis of their socioeconomic status and insurance coverage [9]. Low socioeconomic status has been shown to be an important risk factor for poor clinical outcomes in CF, resulting in higher healthcare costs, hospitalizations and lower quality of life; understanding the socioeconomic effects of the changing demography of CF is essential [10,11]. Ultimately, undiagnosed or misdiagnosed CF patients are left out of the clinical studies that underpin the development of personalized medicines; the same inequity in demographic data is thus reflected in the ultimate market accessibility of the drug.

Indeed, the inaccessibility of advanced diagnostic tests creates a revolving door of poor population representation in genetic databases and research; insights from disease epidemiology and data collected from registries have shown that the CF population has changed over the years, demonstrating a heterogeneity in prevalence [12]. Although lower compared to European countries, CF incidence is present in Asia, Africa, the Middle East and Latin America, its prevalence is underestimated in these regions due to limited and unrepresentative data [5]. This results in the misdiagnosis, or the underdiagnosis, of individuals from these populations and an underreporting of ethnic-specific variations in carrier frequency or penetrance of CFTR variants.

**Figure 1:**
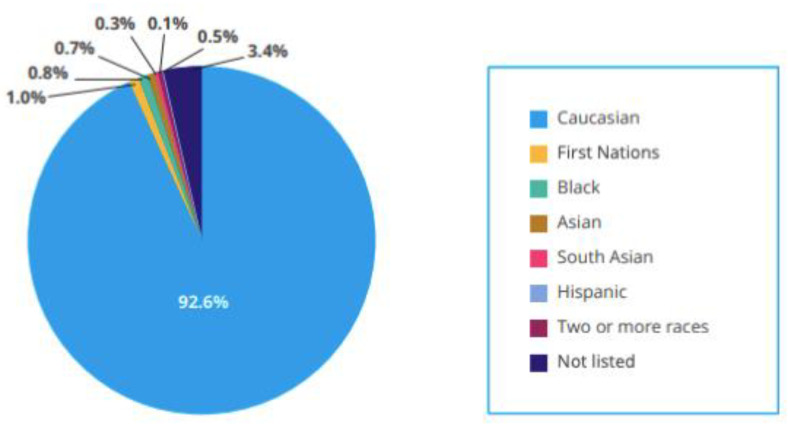
Ethnic distribution of the CF population, taken from the 2018 Canadian CF Registry Annual Data Report [16].

Newborn screening, genetic testing, and counselling support the diagnosis of rare diseases. However, access to such advanced diagnostics, sequencing and the ability to interpret these data is not consistently available across Canada [13,14]. Furthermore, this is exacerbated by (1) a reliance on primary care physicians who do not have the training or access to the resources required to offer these diagnostics; and (2) a reliance on CF experts that are concentrated in urban areas [13,14]. As a result, those with CFTR variants that can be studied or treated fall through the cracks. Furthermore, where they are accessible, minorities are less likely to be detected on prenatal and newborn screening tests due to different frequencies of CFTR variants and the use of DNA panels optimized for European populations [15]. The 2018 Canadian Cystic Fibrosis Registry Report provides the following demographics of identified CF patients, on the basis of race (Figure 1): 

Due to such underdiagnosis and accessibility issues, CF clinical research is biased towards individuals of European descent, and affluent groups are overrepresented. For example, one study showed that in the 19.7% of pharmacology trials in cystic fibrosis between 1999 and 2015 that included any description of race and ethnicity, 94.4% of subjects were non-Latin white subjects [11]. By excluding minorities from clinical trials, we have a limited understanding of CF; these racial disparities have incited criticism for restricting the robust identification of rare diseases and compromising the understanding of patient responses to therapies among certain populations [17,18]. The concern is that these disparities will be perpetuated in the ultimate market accessibility of CF personalized medicines.

### 2.2. Mitigating Strategies

To reduce the risk of widening the health disparity gap in the delivery of personalized medicines, efforts should be made to improve access to advanced diagnostics and clinical research for cystic fibrosis. With CF having a broader clinical and genetic spectrum than previously thought, diagnosis should be supported by altering diagnostic algorithms so as to take into account non-European populations with different CF phenotypes and genotypes. While ensuring transparent processes, interventions should include leveraging prenatal and newborn screening programs, as well as sequencing, that are accessible to all, including those with genetic variants of unclear significance. This will serve to improve the general awareness of CF epidemiology to overcome misdiagnosis and underdiagnosis in minority groups [5]. 

Diversity in genetic databases can be improved through deliberate research to ultimately support equitable access to diagnosis and treatment [19]. For example, Genome BC’s Silent Genomes project aims to reduce access barriers to the diagnosis of genetic diseases in Indigenous children by conducting genomic testing and assessing the economic and healthcare impacts [11]. Additionally, Genome Canada’s “All for One” initiative aims to facilitate the clinical uptake of genome-wide sequencing for rare diseases [20]. In keeping with the principle of non-discrimination and physical accessibility, similar government-initiated efforts ought to be made in marginalized communities in the form of staff training and infrastructure to support the delivery of personalized medicine. Collecting more representative genomic data can improve the ability of researchers to identify new rare gene variations and provide targets for personalized medicines in the future. However, there is a notable ethical concern of exploiting a patient population in data collection that may not have access to the medicine once it is on the market. 

Furthermore, in addition to reporting the race and ethnicity of clinical subjects, pharmaceutical companies and investigators ought to prioritize the inclusion of minority subjects in therapeutic studies of cystic fibrosis [11]. Subject interest in clinical trials is not the cause of this disparity, because minorities are just as willing to participate as their white counterparts [11]. This requires a degree of cultural awareness and sensitivity in crafting research objectives that are aligned with community engagement; investigators ought to consciously design clinical trials to maximize the participation of whom new therapies may be prescribed to [11]. For example, study questionnaires and consent forms should be translated into other appropriate languages and be administered by native-speaking staff when feasible [11]. Ethics boards also need to acknowledge and accept that compensation, including for medical travel, may be required in order to encourage participants with poorer socioeconomic status, and balance this against the concern that compensation that is only adequate for some may be seen as inducement for others. 

In short, the inclusion of minorities in cystic fibrosis diagnostics and studies is necessary to foster equity in accessing the delivery of personalized medicines. The principle of beneficence in bioethics requires adequate infrastructure and training to improve cystic fibrosis diagnosis in marginalized communities [14]. Taken with inclusion in clinical trials, this serves to improve the accessibility of developments in cystic fibrosis therapies and counteract health disparities. 

## 3. Regulatory Oversight and Market Authorization

### 3.1. Challenges 

In Canada, the value of new drugs, including personalized medicines, involves multiple decision factors and several actors. In order to have a drug listed in a public formulary, the first step is the receipt of market authorization; this allows for the sale of a therapy. Post-market authorization allows some patients to access treatments via private health insurance plans or by paying out of pocket. To be publicly accessible, three additional stages are necessary: health technology assessments (“HTAs”); price negotiation; and provincial public formulary inclusion. Thus, before inclusion in the public formularies, health disparities are exacerbated on the basis of insurance coverage, favouring the access to new therapies by the affluent. Often taking years to complete, this can delay access to treatment, resulting in progressive health deterioration [19].

Multiple reviews in the sequential decision-making process lengthens the time for approved personalized medicines to be included in the public formularies—inhibiting public patient access [21]. Canada ranks 15th out of 20 in OECD jurisdictions, taking an average of 27% longer to list new therapies in public formularies [22]. It should be noted, however, that while speed is important, the primary goal of HTA is not accessibility, but cost containment; they ensure that adequate efforts are made to thoroughly review the evidence to make an informed decision about a product’s price [21]. As Health Canada approval and HTA are typically sequential processes, this increases the time in which a drug is only accessible in the private market, creating an equity gap on the basis of socioeconomic status. 

The unique nature of personalized medicines also creates issues in HTA processes, which traditionally rely on methodology that does not translate well into the assessment of rare diseases medicines [23]. The time it takes to review, approve, and include a therapy in public formularies might exceed the prognosis of a life-threatening disease [24]. Further, due to the progressive nature of CF, earliest possible access is necessary so that young patients may benefit before the disease has escalated.

In rare genetic research such as cystic fibrosis, the stratification of patient populations by genetic variants results in smaller sample populations. Combined with variable treatment pathways and poor population representation, the level of uncertainty associated with cost-effectiveness estimates presented to decision-makers increases [25]. Furthermore, many personalized medicines lack available comparator treatments against which to assess safety and efficacy [26]. The lack of long-term safety and durability evidence for personalized medicines, combined with the possible lack of alternative treatment options, makes it difficult to decide whether to offer or choose a personalized medicine as treatment [27]. Furthermore, the pricing of such orphan drugs is primarily driven by the need to recoup significant research and development costs from a small patient population [27]. Due to the high costs of personalized medicine and the use of incompatible methodology for HTA, there is a tendency for cost considerations to take precedence over clinical value, leading to significant delays or blocks to patient access. 

Moreover, mechanisms that trigger post-market reassessment for drugs with uncertain long-term efficacy are lacking [21]. All drugs that receive market authorization in Canada are subject to post-market surveillance by Health Canada and the Public Health Agency of Canada. Given the challenges posed by rare disease research for personalized medicine, Canada has approved these therapies with requirements for post-market surveillance that aim to fill the evidentiary gap [28]. However, the post-market requirements frequently lack transparency and are subject to delays—further reinforcing accessibility issues on the basis of socioeconomic status. 

Finally, Health Canada has acknowledged that emerging technologies, such as gene therapies, may not be suited to the Food and Drug regulatory pathways due to their novelty, complexity and personalized nature [21]. This inhibits incremental improvements in approved personalized medicines because any changes made, including those related to manufacturing or adding new indications, will likely trigger additional regulatory requirements. 

### 3.2. Mitigating Strategies 

A coordinated national approach to reviews and funding decisions is necessary to shorten the time it takes for a personalized medicine to be included in public formularies and align with the principle of financial accessibility. Furthermore, consistency in market authorization, HTA and funding decisions will allow the industry to better predict the process and related timeline, while improving transparency. 

To improve efficiency, a supplemental process for highly specialized or complex drugs could allow parallel reviews by multiple actors [21]. The recommendations for a national approach to pharmacare suggest a Canadian drug agency should be created to evaluate the effectiveness of drugs and negotiate prices [21]. Moreover, regulatory collaboration with other international jurisdictions could also reduce market authorization review times; Health Canada initiatives such as worksharing (i.e., streamlining joint review processes) and the consideration of foreign reviews (i.e., the local use of foreign market authorization) could alleviate delays [29]. The Australian–Canada–Singapore–Switzerland (ACSS) Consortium is a useful example. By creating harmonization amongst the regulatory and decision-making steps, sponsors of eligible drugs could submit applications for concurrent reviews to multiple agencies. This would ultimately reduce the wait time for qualifying patients to access the treatment. 

The complex nature of personalized medicines also calls for the tailoring of the existing approach to valuation. Some mitigating factors already exist: CADTH has provisions for issuing recommendations for reimbursement under conditions including when the new therapy addresses a “significant unmet need” despite uncertainty, such as rare conditions with no alternative treatments and the PMPRB’s threshold for high-cost therapies at USD 150,000 per QALY; however, these cost USD 200,000 for those that are the first effective treatment for an illness [21].

However, differing value conceptions can yield conflicting assessments of the merits of publicly funding particular personalized medicines [30]. Thus, structured value assessments can be designed to reflect societal values, aid decision-making and improve transparency in the process [30]. Beyond life expectancy and the quality of life offered by a drug, many other elements of value have been proposed, including the rarity of condition, the availability of alternatives, closeness to end of life, novelty, curative nature of treatment, societal impacts and the severity of illness. Furthermore, there is a need for transparency and consensus on how various factors are weighed to support greater reliance on value-based healthcare decision-making [21]. Ideally, these factors would be informed by the extent to which Canadian society is willing to trade-off health and non-health benefits. Engaging stakeholder groups, such as patient organizations/advocates and public/private funders, will offer greater clarity and consistency on how funding decisions are made in Canada and manage patient and sponsor expectations about the future public funding of therapies under development.

Issues related to bias ought to be addressed by developing mandatory national registries. Registries, like the comprehensive Canadian CF Registry, help to track uncertainty over long-term safety, durability and adherence to personalized medicines, by providing a mechanism to monitor and gather evidence, and identifying epidemiological trends [20]. The Canadian CF Registry also allows projections on the long-term potential impact of a drug that is useful when considering the value (e.g., current post-market studies on the long-term impact of Ivacaftor and Ivacaftor/Lumacaftor in Canada use the Registry). Concerns inevitably raised about access to information and related risks to privacy are remedied through ensured informed consent. However, ethical issues may also arise if treatment is contingent upon enrollment in a registry; some see it as necessary to inform safety and efficacy data, others see it as coercion [19].

Lastly, a post-market authorization renewal policy could also manage uncertainty relating to long-term safety and durability. A renewal policy would provide regulators with an opportunity to re-evaluate the safety and efficacy of previously authorized drugs; the EMA is an example of a regulatory body that requires manufacturers to submit an application for renewal five years after the original market authorization is granted [31]. CADTH has acknowledged the importance of RWE for HTA and is developing a reassessment framework, as well as considering its use for comparative safety and effectiveness over the long term—however, any such reassessment ought to include patient inputs too [32]. The evidence gathered through post-market surveillance, including RWE, is relevant to regulators, HTAs, payers and prescribers, as uncertainties in safety and efficacy are carried throughout the therapy’s life cycle.

## 4. Implementation into the Healthcare System

### 4.1. Challenges

The issues surrounding the implementation of CF personalized medicines into the healthcare system deals with both the financial and physical accessibility of personalized medicines. First, the readiness and ability of each provincial healthcare system to afford these products will determine whether or not the potential of personalized medicines in CF will be fully realised [23]. Then, the distribution of personalized medicines will determine the equitability of the physical access to these treatments. 

The high costs of personalized medicines will pose a significant financial burden on Canadian public payers in years to come. Traditionally, for treatments like gene therapies, a one-time payment is made up front, despite uncertainty about the duration of the drug’s effect, long-term safety risks and associated additional healthcare spending [23]. It is inevitable that personalized medicines will increase the overall costs of healthcare, even if given selectively [33]. For example, Ivacaftor is currently licensed in the United Kingdom for those over 6 years of age, available for approximately 370 patients, and costs GBP 67 million per year, collectively. In Canada, CADTH approved KALYDECO^®^ (Ivacaftor) for 146 CF patients under the condition of a substantial reduction in price; at the submitted price of CAD 306,600 per year, the incremental cost per quality-adjusted life year (QALY) for Ivacaftor is approximately CAD 926,776 and could be as high as CAD 4.6 million per QALY [34]. Some suggest a patient would do better with a full-time private CF nurse than with Ivacaftor [35]. However, it would be wrong if the patients who volunteered their time and bodies for studies, and whose families raised funds, cannot benefit from the drug—for example, despite its philanthropically funded early stage development, KALYDECO^®^ was distributed by drug companies for profit, precluding access for patients without the financial resources [36].

It is equally wrong if some patients cannot access a treatment that is available to others. The delay in the inclusion of personalized medicines in public formularies allows those with financial resources or private insurance to access these medicines before the uninsured. This favours groups with the resources to fund research and buy access to high-priced therapies not available in the public system [37]. Furthermore, the prospect of underfunded therapies could contribute to disparities in health; for example, four gene therapies approved for the EU market were withdrawn due to a lack of funding (see Glybera), forcing patients to turn to private means or appeal to the government or manufacturer for exceptional access [38]. Furthermore, reliance on crowdsourcing raises its own set of ethical questions [39]—does it favour campaigns conducted by those with wider networks, better marketing skills, or fluency in the dominant language? Similar ethical issues are raised by attempts to “level the playing field” via lotteries, for example, the distribution of Zolgensma in the U.S. [17]. Importantly, do these avenues obscure issues of justice in public healthcare coverage?

The high costs associated with health behaviour changes are another source of disparity in the benefits personalized medicine has the potential to deliver. Medical problems influenced by health behaviours disproportionately affect patients in lower socioeconomic strata; for example, minority children are more likely to be exposed to air-pollution and pesticides, contributing to abnormal pulmonary function and respiratory tract disease [40]. The disparity reflects the degree to which personal finances and the government allocation of resources affect patient access to healthy foods, exercise facilities and other services related to healthy behaviours. The patients’ ability to make meaningful changes to their health behaviours will impact the efficacy of personalized medicines—this hinges on their access to social resources. 

**Figure 2:**
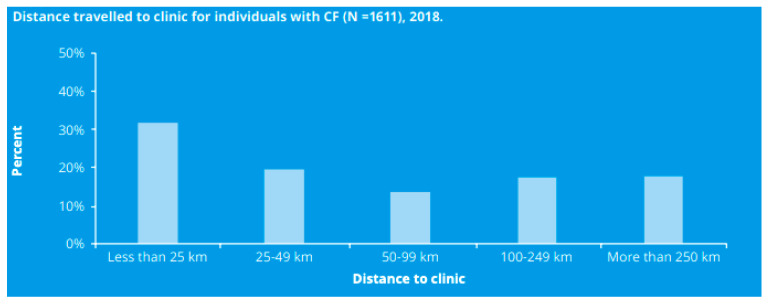
Distance travelled to clinic for individuals with CF, taken from the 2018 Canadian CF Registry Annual Data Report [16].

The physical accessibility efforts in the delivery of personalized medicine will dictate its equitable incorporation in the Canadian healthcare system. Geographical proximity and the physical accessibility of health services are strongly associated with treatment adherence and clinical outcomes. The figure below, taken from the 2018 Canadian Cystic Fibrosis Registry, indicates the percentage of patients that travel certain distances in order to have access to a CF clinic (Figure 2):

It is likely that access to such treatments will be concentrated in large urban centres due to the presence of CF experts, depending on the complexity of its administration [19]. As such, many patients and their caregivers may need to travel in order to receive treatment and remain on the site for an extended period of time, on their own money. 

Furthermore, patient access will depend on their province or territory of residence. While this is not an issue exclusive to the delivery of personalized medicines, it is exacerbated by their high costs: “constraints based on the availability of infrastructure needed for provisioning become compounded by the fragmented multi-payer landscape of reimbursement mechanisms across Canada” [19]. This raises questions about equity at the federal level and leaves room for inconsistency across national borders.

### 4.2. Mitigating Strategies 

To manage healthcare budgets, additional controls are needed when high-cost therapies are funded in Canada. Another layer of review for drugs that are expected to exceed some spending threshold would be an option; this requires careful design, because it can disadvantage one-off treatments with high costs and lead to suboptimal access [41]. In the United Kingdom, the National Institute for Health and Care Excellence (“NICE”) introduced enhanced scrutiny, price negotiations and the potential for restricted access for therapies expected to exceed a threshold of GBP 20 million in annual spending in any of the first three years [42]. Furthermore, an emphasis on the development of alternative treatment pathways is desirable; this is because individual responses to different drugs can be quite varied, but also because competition generally helps brings prices down. Additionally, competition should arrive in the form of generics as the original modulators come off patent (which should be in the late 2020s).

To mitigate issues of physical accessibility, support for medical travel can alleviate access challenges for patients in remote and rural regions. All provinces, except Alberta and New Brunswick, offer some form of medical travel assistance to residents. However, the remaining variations in availability and the resourcing of government travel programs can contribute to uneven access [43]. In addition to financial costs, medical travel removes patients from their cultural and community support [19]. Considering the existing inaccessibility of healthcare in Indigenous reserves, combined with a historical distrust of colonial institutions, medical travel is not a perfect solution. However, these challenges are less pronounced for in vivo therapies and may facilitate the creation of more treatment locations [19]. This limitation to physical accessibility could also be alleviated through outreach care programs and telehealth services. Since COVID-19, virtual care has become regular practice; services such as Gene Matters provide remote genetic testing [44].

To address jurisdictional disparities in the delivery of healthcare, pan-Canadian programs and frameworks could be considered to resolve challenges relating to differences in the outcome and timing of reimbursement decisions across provinces and territories. For example, the Canadian Fabry Disease Initiative produced a national database and tested a cost-sharing agreement between the federal and provincial governments for the expensive treatment of a rare disease to coordinate a framework for access [45]. Lessons from the CFDI could better prepare decision-makers for the influx of expensive therapies for rare diseases. 

Other payment plan options include price caps and volume caps to limit expenditures to an agreed upon total number of annual payments or treatment courses, cost-free after that point for additional treatment or price-volume agreements to ratchet down prices in a stepwise manner at various purchase thresholds [46]. Subscription models are also possible (i.e., a plan pays a fixed annual fee for unlimited drug access); whilst in the United States, subscriptions are used where insurers struggle to absorb costs, such as hepatitis C medication [47].

Furthermore, special criteria and dedicated funds can carve out resources for supporting high-cost therapies. Research from Canada and other jurisdictions suggests public support for special treatment for severe illnesses with a lack of treatment options, but not only on the basis of rarity [48]. For example, the federal government’s Pharmacare Budget 2019 intends to provide funding in 2022–2023 of up to CAD 1 billion over two years to improve access to high-cost therapies for rare diseases, with potential for ongoing annual CAD 500 million funding after that [49]. This is a promising option for similarly publicly funded healthcare and pharmacare systems. Lessons from international experience with orphan drug policies indicate that national guidelines or programs relating to funding and HTA are a favourable strategy to incorporate personalized medicine into the healthcare system [50].

Lastly, alternative provision models are emerging in response to market conditions. Public research investments are one means to reduce costs by extending public influence over made-in-Canada therapies through licensing agreements, rather than the selling of patents. Furthermore, moving to not-for-profit development and provision models can increase affordability [17]. Social entrepreneurship models can be used by companies seeking to deliver social returns, avoiding the issue of halting the development of “non-profitable” therapies. Additionally, philanthropic undertakings could also play a role in providing affordable access. However, this is placing public responsibility into the hands of corporations. 

## 5. Mitigating Strategies at Each Stage: Summary

1. Clinical Research and Diagnostic TestingImproved access to advanced diagnosticsLeveraging prenatal and newborn screening programs to overcome underdiagnosis in minority groupsSequencing with ensured consentDeliberate efforts to improve diversity in genetic databasesTraining and infrastructure in marginalized communities to collect representative dataDeliberate efforts to improve diversity in clinical research Report on race/ethnicityCulturally sensitive trial designs and objectivesLanguage-accommodating consent forms and questionnaires2. Regulatory Oversight ad Market AuthorizationCoordinated national approach to review and fundingSupplemental process for complex drugsPharmacare drug agency to evaluate price and effectivenessInternational regulatory collaboration: worksharing, consideration of foreign reviews, etc.Tailoring valuation to the nature of PMsStructured value assessments that consider the rarity of conditions, the availability of alternatives, closeness to end of life, novelty, curative nature of treatment, societal impact and severity of illnessTransparency in decision making for predictabilityManaging long-term uncertaintyMandatory national registriesPost-market authorization renewal policies3. Implementation into the Healthcare SystemManaging healthcare budgetsAdditional layer of review: enhanced scrutiny, price negotiations and potential for restricted access for therapies that are expected to exceed a price threshold in annual spending for the first × amount of yearsPayment plan options: price caps, volume caps, stepwise, subscriptionsImproving physical accessibilityNational support for medical travelOutreach care programsTelehealth servicesManaging jurisdictional disparityPan-Canadian programs/frameworks for reimbursement decisionsAlternative provision modelsLicensing agreementsSocial entrepreneurial modelsPhilanthropic undertakings

## 6. Conclusions

The introduction of personalized medicines into Canada’s healthcare system can improve patient care by addressing respective gene variants and treating their resulting symptoms. However, the nature of such treatments, like its small target population and uncertain durability, make it difficult to justify within current regulatory frameworks. This requires an accommodation of the unique benefits of personalized medicines to fill the treatment void in a nationally harmonized and efficient way. Furthermore, the poor racial representation currently reflected in CF diagnosis, studies and biobanks will inevitably be perpetuated in the delivery of personalized medicines, widening the health disparity gap. Conscious efforts must be made to include minorities in research studies, diagnostic tests, and data/biobanks to ensure the accuracy and accessibility of personalized medicines. 

However, due to the complexity of equitably introducing personalized medicines into the Canadian healthcare system, efforts should not be lost on holistically serving the CF population. As there are and will continue to be individuals with CF without access to CFTR modulators, there is greater need for research and medical developments in the area of symptomatic therapies that target and meet the needs of those populations. CFTR modulators are not effective in severe cases of CF where lung damage cannot be reversed. Furthermore, alternative treatments that are effective, and yet do not rely on an individual’s underlying genotype, could benefit a wider number of CF patients, and could include novel approaches using patient-derived model systems that support clinical improvements, stem cell technologies or gene editing [51]. Our recommended multifaceted approach may serve to provide solutions to ensure that people with CFTR variants can benefit from innovative personalized medicines and those that cannot have alternative treatment pathways. 

## Data Availability

Not applicable.
